# Augmented reality navigation in open surgery for hilar cholangiocarcinoma resection with hemihepatectomy using video-based in situ three-dimensional anatomical modeling: A case report

**DOI:** 10.1097/MD.0000000000008083

**Published:** 2017-09-15

**Authors:** Rui Tang, Longfei Ma, Canhong Xiang, Xuedong Wang, Ang Li, Hongen Liao, Jiahong Dong

**Affiliations:** a Department of Hepatopancreatobiliary Surgery, Beijing Tsinghua Changgung Hospital; b Department of Biomedical Engineering, School of Medicine, Tsinghua University, Beijing, China.

**Keywords:** augmented reality navigation, hepatectomy, hilar cholangiocarcinoma

## Abstract

**Rationale:**

Patients who undergo hilar cholangiocarcinoma (HCAC) resection with concomitant hepatectomy have a high risk of postoperative morbidity and mortality due to surgical trauma to the hepatic and biliary vasculature.

**Patient concerns:**

A 58-year-old Chinese man with yellowing skin and sclera, abdominal distension, pruritus, and anorexia for approximately 3 weeks.

**Diagnoses:**

Magnetic resonance cholangiopancreatography and enhanced computed tomography (CT) scanning revealed a mass over the biliary tree at the porta hepatis, which diagnosed to be s a hilar cholangiocarcinoma.

**Intervention:**

Three-dimensional (3D) images of the patient's hepatic and biliary structures were reconstructed preoperatively from CT data, and the 3D images were used for preoperative planning and augmented reality (AR)-assisted intraoperative navigation during open HCAC resection with hemihepatectomy. A 3D-printed model of the patient's biliary structures was also used intraoperatively as a visual reference.

**Outcomes:**

No serious postoperative complications occurred, and the patient was tumor-free at the 9-month follow-up examination based on CT results.

**Lessons:**

AR-assisted preoperative planning and intraoperative navigation might be beneficial in other patients with HCAC patients to reduce postoperative complications and ensure disease-free survival. In our postoperative analysis, we also found that, when the3D images were superimposed 3D-printed model using a see-through integral video graphy display device, our senses of depth perception and motion parallax were improved, compared with that which we had experienced intraoperatively using the videobased AR display system.

## 1 Introduction

Cholangiocarcinoma, which can involve the intrahepatic or extrahepatic biliary ducts and their confluences, accounts for approximately 15% of primary hepatic tumors worldwide.^[1]^ Resection is the most effective treatment for hilar cholangiocarcinoma (HCAC),^[2,3]^ which is the most common type of cholangiocarcinoma.^[3–5]^ However, HCAC resection is challenging due to difficulty accessing structures in the porta hepatis, the proximity of major hepatic vasculature, and variation in biliary anatomy. Patients receiving curative HCAC surgery with concomitant hepatic resection suffer greater postoperative morbidity and mortality than patients without hepatic resection, but they also exhibit higher 5-year survival as a result of the removal of invading tumor cells in the adjacent liver parenchyma.^[4,6,7]^ The development of techniques for attaining disease-free margins while minimizing damage to the bile ducts and hepatic vasculature during concomitant resection for HCAC is, therefore, critical for improving patient outcomes.

Augmented reality (AR) technology seeks to improve surgical outcomes by creating organ-level minimally invasive conditions. In AR, computer-generated 3-dimensional (3D) images based on preoperative medical imaging data are superimposed on real-time intraoperative video to improve the visualization of anatomical structures that are otherwise difficult to distinguish,^[8–10]^ thereby enhancing surgical precision. Previous reports have demonstrated the reliable use of AR technology for various types of hepatic resection in robotic,^[8]^ laparoscopic,^[10]^ and open surgical procedures.^[11,12]^ In our current report, we demonstrate the use of AR technology in open radical HCAC surgery with concomitant hepatic resection in a patient with obstructive HCAC, and assessed recovery over a 9-month follow-up period.

## 2 Case report

### 2.1 Presentation and Diagnosis

**Figure 1:**
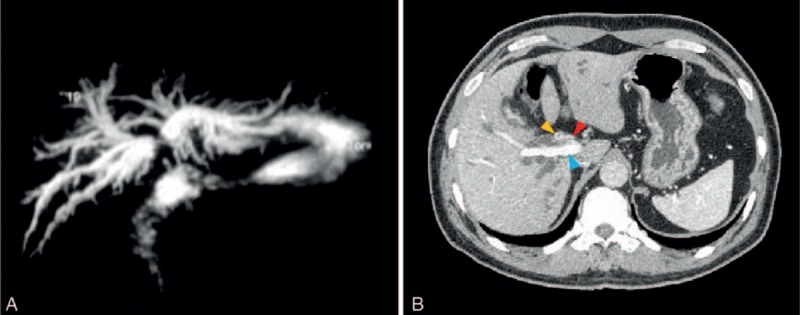
Imaging results for a 58-year-old man who presented with yellowish skin and sclera, abdominal distension, pruritus, and anorexia for 3 weeks. (A) Magnetic resonance cholangiopancreatography and (B) enhanced computed tomography showed a hilar cholangiocarcinoma (red arrow: right hepatic artery; yellow arrow: tumor; blue arrow: right portal vein).

A 58-year-old man presented to our hospital with yellowing skin and sclera, abdominal distension, pruritus, and anorexia for approximately 3 weeks and a total weight loss of 5 kg. The clinical examination found no obvious abdominal abnormalities. Routine blood analysis showed that complete blood cell counts and coagulation markers were within the reference ranges. An analysis of liver function and tumor markers showed the following serum levels: alanine transaminase, 68.2 U/L; aspartate transaminase, 40.3 U/L; alkaline phosphatase, 223.8 U/L; gamma glutamyl transaminase, 11.5 U/L; total bilirubin, 429.1 μmol/L; direct bilirubin, 352.6 μmol/L; CA19–9 <2.00 U/mL; carcinoembryonic antigen, 3.03 ng/mL; and alpha fetoprotein, 2.90 ng/mL. Magnetic resonance cholangiopancreatography (MRCP) and enhanced computed tomography (CT) scanning revealed a mass over the biliary tree at the porta hepatis, and a diagnosis of HCAC with intrahepatic cholangiectasis was made (Fig. 1).

Percutaneous transhepatic biliary drainage (PTBD) of the left and right anterior intrahepatic biliary ducts was performed on days 3 and 12 of treatment, respectively. Analysis of liver function immediately prior to surgery showed the following serum levels: alanine transaminase, 110 U/L; aspartate transaminase, 86.5 U/L; alkaline phosphatase, 169 U/L; gamma glutamyl transaminase, 193.0 U/L; total bilirubin, 174.9 μmol/L, and direct bilirubin, 147.8 μmol/L. The patient was scheduled for curative HCAC resection on day 35 of treatment. Written, informed consent was obtained from the patient before undergoing the surgical intervention, and all procedures were performed in accordance with the Declaration of Helsinki regarding the ethical principles for medical research involving human subjects.

### 2.2 Perioperative procedures

Preoperative 3D image reconstruction of the liver parenchyma, hepatic arteries (HAs), portal vein (PV), hepatic veins (HVs), biliary tree, gallbladder, inferior vena cava, HCAC tumor, and PTBD tubes was performed using the Materialise Mimics, version 16.0, software (Leuven, Belgium) based on the 2-dimensional (2D) CT data, as described previously.^[8]^ A CT slice thickness of 0.625 mm was used for the reconstruction. The patient's intrahepatic cholangiectasis was significant, so biliary structures were clearly visible in the CT images. The MRCP data were not used for 3D image reconstruction, but MRCP images were used as a surgical reference and for amending the CT-based reconstruction. Virtual surgery planning for perihilar resection and left hemihepatectomy was performed using the IQQA-Liver program (EDDA Technology, Princeton, NJ).

Cholangiocarcinoma typically invades either along the biliary duct wall or into the liver parenchyma, but few reports exist describing evidenced-based approaches for HCAC resection. Our previous experience with HCAC resection has shown us that the biliary duct should be resected around its axis, leaving a tumor margin of ≥5 mm.^[13]^ Sakamoto et al^[14]^ reported that, for nodular-infiltrating or diffusely infiltrating HCAC tumors, the mean length of submucosal extension was 6 mm, and the length was <10 mm in 83% of cases. Sasaki et al^[15]^ reported that, among 19 HCAC patients with a median biliary duct margin of 11.4 mm (range, 6.5–17.0 mm), only 1 case was found to have a cancer-positive margin. With regard to hepatectomy, Endo et al^[16]^ reported that the optimal resection line for left lobe hepatectomy crosses the bifurcation of the right anterior and right posterior branches of the right portal vein, which they referred to as the “P point.”

**Figure 2:**
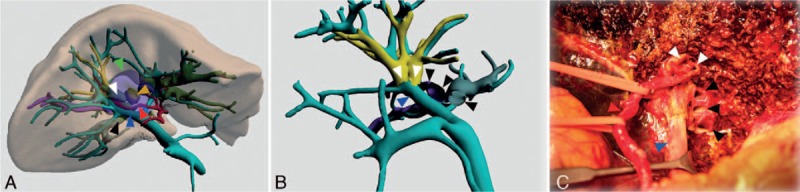
Reconstructed 3D images. (A, B) Reconstructed images of the liver, hepatic vasculature, biliary ducts, and tumor viewed from the inferior aspect showing the tumor (yellow arrow), resection margin border (green arrow), hepatic artery (red arrow), portal vein (blue arrow), right anterior bile duct (white arrow), and right posterior bile duct (black arrow). (C) Intraoperative view of the hilum showing the hepatic artery (red arrow), portal vein (blue arrow), and right anterior bile ducts (white arrow), and right posterior bile ducts (black arrow). The 5 hepatic ducts are in accordance with the preoperative surgical plan under the intraoperative video-based AR navigation. AR = augmented reality.

**Figure 3:**
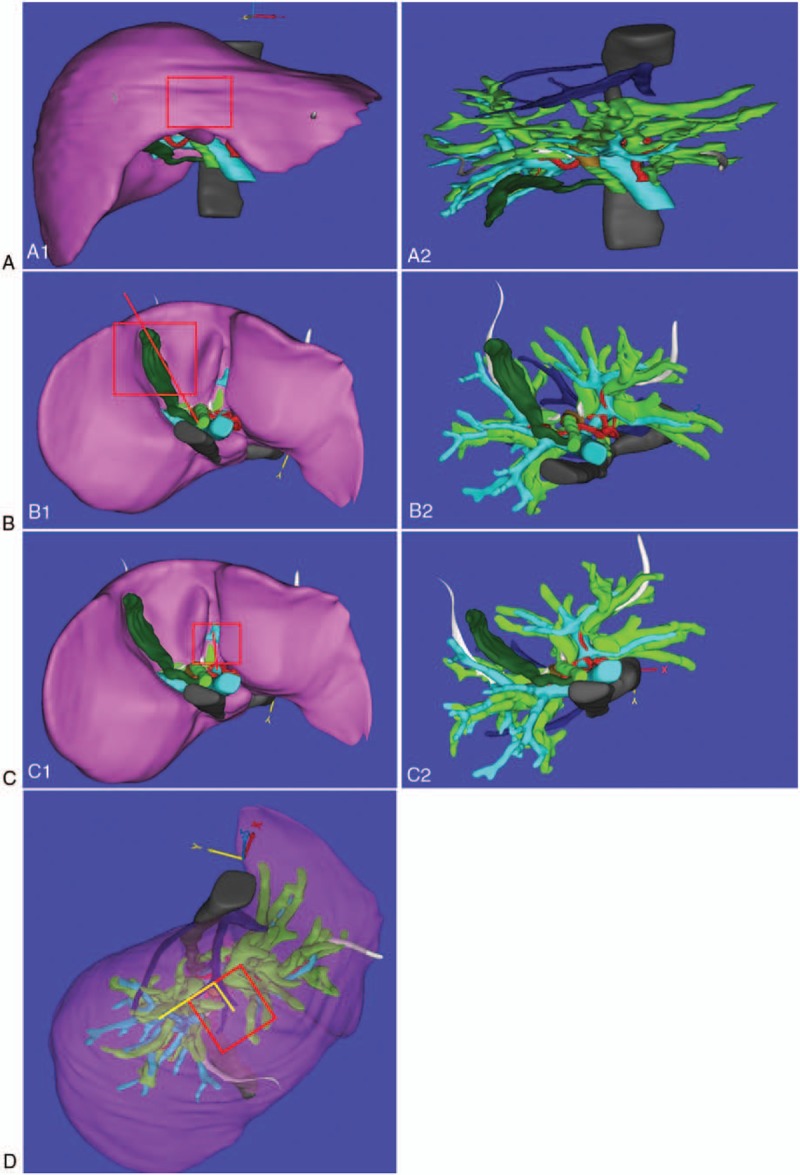
The locations of the anatomical landmarks. (A1, A2) The midpoint between the percutaneous transhepatic biliary drainage tubes along the inferior border of the right lobe, (B1, B2) gallbladder fossa, (C1, C2) portal vein sagittal section, and (D) bifurcation of the middle hepatic vein were used as anatomical landmarks for aligning the reconstructed 3D images of the patient's liver with those of the involved biliary tree and adjacent hepatic vasculature.

To ensure R0 resection margins for our case, we estimated a resection margin of 15 mm, which passed exactly through the P point. This approach would sever 5 right intrahepatic biliary ducts, but preserved the trunk of the middle HV, right HA, and PV (Fig. 2). The application of the software AR Liver Xin (X-Real, Peking, China) was used to generate the AR intraoperative video. Four 3 × 3 cm 2D code patterns were placed at the gallbladder fossa, PV sagittal section, bifurcation of the middle HV, and midpoint between the PTBD tubes along the inferior border of the liver for image registration (Fig. 3) using an intraoperative digital video camera and an iPad computer (Apple Computers, Cupertino, CA).

### 2.3 Three-D printed model experiment

Surgeons who have used video-based AR technology, such as that employed for intraoperative navigation in our study, often report diminished depth perception and motion parallax,^[17–19]^ with which we concur. Therefore, immediately following surgery, we evaluated the imaging characteristics of autostereoscopic see-through display based on integral videography (IV), and compared them with those of the video-based display method that we had used for intraoperative navigation. A 3D-printed model of the patient's biliary tree and adjacent vasculature was produced preoperatively from the reconstructed 3D images using an Object Connex 350 3D Printer (Stratasys, Eden Prairie, MN). This model was used intraoperatively as surgical reference. The reconstructed 3D images were also overlaid onto the 3D-printed model using an IV image overlay device equipped with a microconvex-lens array and a half-silvered mirror. An optical tracking probe was calibrated using the pivot calibration method, and the probe and an optical tracker (Polaris Spectra Position Sensor, Waterloo, Ontario, Canada) were used to establish 4 vessel bifurcations on the 3D model as anatomical landmarks for image registration. Rigid registration was performed, aligning these 4 points with the corresponding locations in the reconstructed 3D images, using IV and an LCD display.

### 2.4 Laparotomy and tumor assessment

Following laparotomy using the Kocher abdominal incision, the lymph node 13 was removed. The common bile duct was severed at the superior border of duodenum, and lymph nodes 8 and 12 were removed. The common HA, gastroduodenal artery, left HA, middle HA, and the anterior and posterior branches of the right HA were separated. The trunk of the PV and the proximal segments of the left PV and the anterior and posterior branches of the right PV were exposed. The tumor was located at the bifurcation of the common and left hepatic ducts, and tumor invasion was visible at the primary bifurcation of the PV only.

### 2.5 Tumor resection and hemihepatectomy

**Figure 4:**
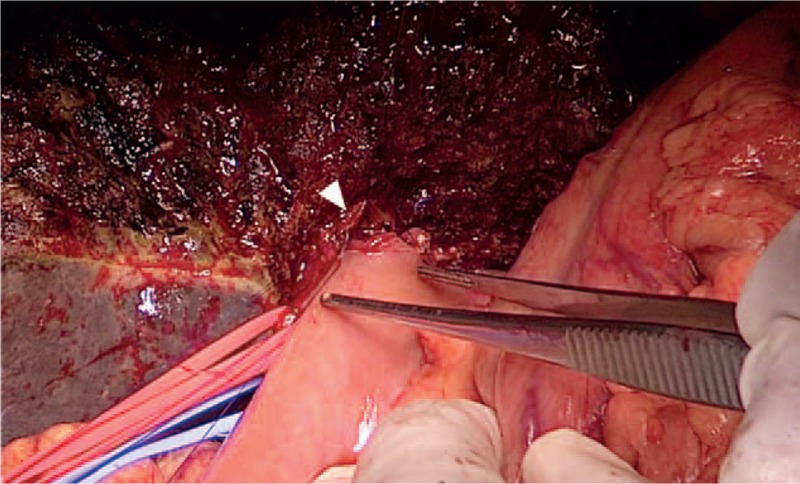
Intraoperative view of the hepaticojejunostomy. The efferent arm of the jejunum was surgically joined with the liver at the bile ducts (white arrowhead). The flow of bile into the small bowel was restored by jejunojejunostomy, creating the anastomosis of the afferent arm and the intact jejunum.

Given the location and local invasion of the tumor, the surgical team decided to proceed with the perihilar HCAC procedure with PV resection and reconstruction and left hemihepatectomy. Following the placement of the 4 2D code patterns, the middle HV was located by ultrasound scanning, and we adjusted the 2D code patterns to align the real and virtual images during registration at slightly different orientations. The following steps were performed with the assistance of the video-based AR navigation system and ultrasonography: incising the liver parenchyma along the left side of the middle HV; severing the right PV; estimating the boundary of the tumor with regard to the HAs and biliary ducts; determining the drainage basins of the PTBD tubes, and incising 1.5-cm tumor margins in the adjacent parenchyma. The tumor; involved biliary ducts; involved PV segment; liver segments 1, 2, 3, and 4; and parenchyma margins from liver segment 5 were removed. The trunk of the PV and right PV were sutured together for vascular reconstruction. Hepaticojejunostomy was performed, in which the 5 exposed biliary ducts were surgically fused into 1 common trunk for bilioenteric anastomosis (Fig. 4).

**Figure 5:**
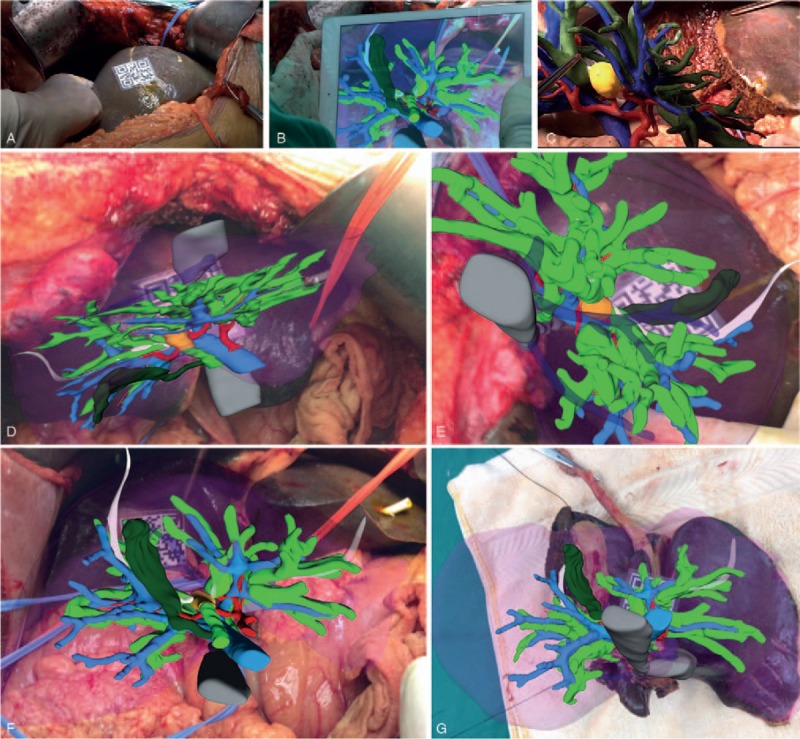
AR-assisted intraoperative navigation. (A) The 2D code pattern (3 × 3 cm) is visible on the anterior surface of the right lobe. (B) After completing registration, the reconstructed 3D images were overlaid onto the image of the surgical field using video-based display. (C) The hand-held 3D-printed model was also viewed by surgeons with the naked eye to assist intraoperative navigation. (D–F) The previous registration of the anatomical landmarks in reconstructed 3D images with those in the real organ at different orientations allowed the AR navigation system to display the virtual images in the approximate in situ orientation on iPad computer. (G) Safe surgical margins were verified by AR on the resected left-lobe specimen. AR = augmented reality.

The resection line established in the preoperative planning was not changed intraoperatively, demonstrating the effectiveness of AR for preoperative planning. During surgery, registration became unstable periodically as the 2D code patterns moved. Therefore, ultrasound scanning was used to visualize the internal hepatic structures for 3D image correction, and registration was repeated. This caused AR navigation to be a discontinuous procedure. Following left hemihepatectomy, the biliary ducts in the reconstructed 3D images of the AR system and 3D-printed model aligned approximately with those in the preserved right-lobe liver. Safe surgical margins were verified by AR on the resected left-lobe specimen (Fig. 5).

### 2.6 Simulated in situ see-through display for HCAC resection

**Figure 6:**
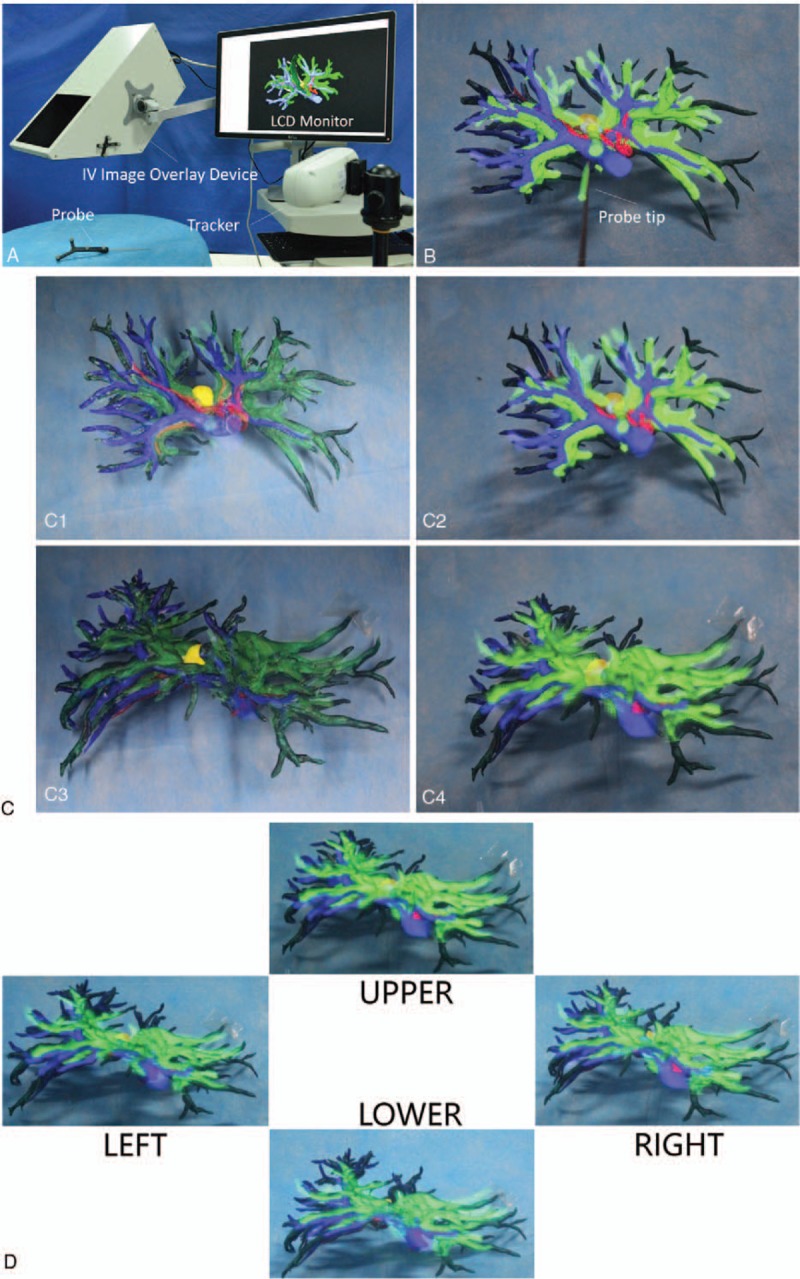
Evaluation of the imaging characteristics of autostereoscopic see-through display using IV. (A) The patient's reconstructed preoperative 3D images were superimposed onto the 3D-printed model using IV overlay device, and LCD monitor. (B) Four vessel bifurcations in the 3D model were designated as anatomical landmarks using an optical tracker and probe. (C1–C4) Rigid registration was performed, aligning these 4 points with the corresponding locations in the reconstructed 3D images. (D) Peering through the viewing window of the IV image overlay device, the surgeons viewed the 3D model and in situ superimposed 3D images from 4 slightly different orientations to estimate whether depth perception and motion parallax were improved, compared with that which they had experience using the video-based display method for intraoperative navigation.

Following surgery, the reconstructed 3D images of the patient's biliary tree and hepatic vasculature were superimposed upon on the 3D-printed model using the IV image overlay device described above, and the autostereoscopic images were viewed through the viewing window of the IV device with the naked eye (Fig. 6). By moving our heads slightly, we viewed the fused in-situ 3D images from 4 slightly different orientations, and found that our senses of depth perception and motion parallax were improved, compared with that which we had experienced intraoperatively using the video-based AR display system. This is the first report of the application of IV in hepatobiliary surgery.

### 2.7 Pathology and follow-up

The results of the histopathological analysis of the resected tumor showed a Bismuth–Corlette classification of type IIIb, an American Joint Committee on Cancer Tumor-Node-Metastasis Stage of cT3N0M0, and an International Bile Duct Carcinoma Cooperative Group classification of B3 L, T2, PV0, HA0, 62.8%, N0, and M0. The pathology confirmed that the resection margin border was HCAC-negative. The distance between the tumor and surgical margin is 1.2 cm of liver parenchyma and 0.4 cm of bile ducts in ours case. The patient was discharged 21 days after surgery, with no serious postoperative complications (Clavien grade I), with the exception of a 2-cm section of the laparotomy incision, which healed poorly. At the 9-month follow-up examination, enhanced CT scanning revealed no sign of tumor recurrence.

## 3 Discussion

We used video-based AR display technology to assist surgical resection of HCAC and concomitant left hemihepatectomy. The patient's enhanced CT and MRCP data were used to reconstruct computer-generated 3D images of the liver. The 3D images were optimized by aligning blood vessel bifurcations in the 3D images with corresponding structures in a 3D-printed model of the tumor, involved biliary ducts, and hepatic vasculature. These 3D images were overlaid onto video images of the surgical field for preoperative planning and intraoperative AR-assisted navigation during perihilar HCAC and semi-hepatic resection. The absence of postoperative complications and disease-free recovery at the 9-month follow-up for our patient suggest that our video-based AR navigation system might be useful for improving surgical outcomes in HCAC patients, and the results of the 3D model experiment warrant further investigation into the use of IV-based see-through AR display technology for open hepatobiliary surgery.

Although intraoperative ultrasonography and static preoperative magnetic resonance imaging (MRI) and CT images are important aids to surgeons for locating hepatic and biliary tumors, ensuring the maintenance of biliary function through careful placement of incisions for liver surgery is challenging, and estimating the amount of tissue required for tumor-free margins is equally difficult. Considering the poor survival rate for HCAC resection with hepatectomy, the development of more precise liver surgery techniques is critical to improving clinical outcomes. A number of investigators have reported the successful use of intraoperative AR technology for liver surgery.^[8,12,20]^ These AR-navigated procedures included hepatic segmentectomy (with and without robotic assistance), hepatectomy, lobectomy, and local resection in open transabdominal surgery^[21–23]^ and laparoscopic segmentectomy using a transthoracic approach,^[20]^ with no serious postoperative complications. Our findings and those of these previous investigations warrant future investigations of AR-assisted navigation for enhancing the precision of liver surgery and improving surgical outcomes.

The major limitation of AR is the inability of the rigid registration of 3D images to intraoperative video images to compensate for deformation of the liver and diaphragm movements in the patient during surgery, which causes the locations of the anatomical landmarks and other liver structures in the patient to change in real time. Substantial liver deformation was not observed in our patient, and the postoperative overlay of the reconstructed 3D images of the biliary ducts aligned approximately with those that were severed in the resected left lobe specimen. Both interactive and automatic registration systems have been developed for hybrid operating rooms with CT imaging instrumentation, which allow 2D image acquisition, 3D image reconstruction, and 3D image to intraoperative video registration to be repeated periodically to adapt to changes in the orientation and spatial dimensions of the liver.^[24,25]^ Investigations of the use of these systems for liver surgery in humans have not been reported.

The positioning of the 2D code on the surface of the liver (Fig. 4) for intraoperative registration limited the effectiveness of our AR navigation method to some extent because the code itself covered a portion of the surgical field. We speculated that this relatively flat surface of the liver would be less affected by organ deformation. At times, however, the line of sight of the intraoperative camera was obstructed by the surgeon's hands or surgical instruments, which consequently reduced the accuracy of the overlaid 3D images, as described previously.^[26]^ We investigated the use of smaller surface areas for the 2D code during preoperative planning, before selecting the 3 × 3 cm coding pattern. The use of smaller coding pattern areas caused a trembling effect on the images in the video display that impaired visual acuity, demonstrating the need for robust registration mechanisms for AR navigation systems. Improvements in the sensitivity of intraoperative video cameras might improve the effectiveness of AR navigation by allowing smaller surface areas for the 2D coding pattern.

## 4 Conclusion

We used enhanced CT and MRCP data to generate 3D images of the hepatic hilar structures of the patient with HCAC using AR technology for preoperative surgical planning and intraoperative navigation during open tumor resection and hemihepatectomy. The disease- and complication-free recovery of the patient over a 9-month follow-up warrant additional investigations in the use of our AR method for reducing postoperative complications and improving clinical outcomes in patients undergoing curative surgery for HCAC. To our knowledge, this is the first report of the use of AR navigation for HCAC resection.
